# Evolution of instrumental flavor profiles in pork with salted vegetable during frozen storage revealed by GC-IMS, E-nose, and E-tongue

**DOI:** 10.3389/fnut.2026.1880506

**Published:** 2026-07-27

**Authors:** Menggong Si, Mengyang Liu, Zhongqing Yang, Xiao He, Jiawei Song, Ronghua Xu, Zhihong Qiao, Li Zhu

**Affiliations:** 1 Tourism College, Beijing Union University, Beijing, China; 2 College of Applied Science and Technology, Beijing Union University, Beijing, China; 3 Faculty of Innovative Hospitality Management, Macau University of Tourism, Macau, China; 4 Beijing Key Laboratory of Bioactive Substances and Functional Foods, Beijing Union University, Beijing, China

**Keywords:** E-nose, E-tongue, flavor, GC-IMS, pork with salted vegetable

## Abstract

This study investigated the impact of storage duration on the flavor profile of a representative prepared dish, pork with salted vegetable (PSV). E-nose results indicated that the intensity of organic compounds, aromatics, and pungent gases increased with prolonged storage. E-tongue analysis revealed that fresh PSV exhibited the highest sourness and umami, which gradually diminished, while bitterness intensified. A total of 79 volatile compounds were identified, including 33 aldehydes, 8 alcohols, 16 ketones, 10 esters, 1 pyrazine, 1 furan, 3 terpenes, and 7 unidentified compounds. ROAV analysis suggested that several aldehydes and ketones, including pentanal, hexanal, heptanal, n-octanal, n-nonanal, 3-methylbutanal, 2-methylbutanal, 2-phenylethanal, 1-octen-3-one, and 3-penten-2-one, were potential aroma contributors in fresh PSV. Using a Partial Least Squares Discriminant Analysis (PLS-DA) model, 19 compounds with Variable Importance in Projection (VIP) scores greater than 1.0 were identified, mainly aldehydes, ketones, alcohols, and esters. These changes may be associated with lipid oxidation and thermal reaction pathways, although further sensory and chemical validation is required. The experimental results provide instrumental evidence for understanding flavor variation in PSV during storage and may support future studies on flavor quality evaluation in similar prepared dishes.

## 1 Introduction

Food flavor primarily refers to the overall perception of flavor compounds by human senses and serves as an important evaluation index during food processing and storage (1). For many restaurant-sold prepared foods, due to the complexity and time-consuming nature of their production processes, a common practice is to prepare them in advance, followed by freezing, thawing, and reheating. This cooking and preparation process poses a key challenge to preserving the flavor of the dishes (2). Flavor changes in prepared foods during processing and storage directly affect dish quality, consumer satisfaction, and sales (3, 4).

Pork with Salted vegetable is a representative traditional dish among meat-based prepared foods. It is widely favored by consumers for its tender meat texture, rich yet non-greasy feel, and unique flavor profile (5). Freshly cooked PSV exhibits a strong meaty aroma and fruity notes. Its flavor molecular composition is primarily composed of alcohols, aldehydes, and terpenes. Alcohol compounds, such as 1-octen-3-ol, possess pleasant odors. Aldehydes, like 2-methylbutanal, are key volatile aroma components in PSV and represent characteristic flavors from meat fat. Terpenes often present woody, fruity, and floral aromas, contributing significantly to the overall flavor (6). As the prepared dish undergoes refreezing, thawing, and reheating, the meaty aroma diminishes, while fatty, sour, and metallic off-flavors emerge (5, 7). This decline in consumer experience is attributed to the development of “warmed-over flavor” (WOF) during reheating. WOF mainly originates from aldehydes and ketones produced by lipid oxidation, such as 1-octen-3-one, nonanal, and hexanal (8–10). Therefore, analyzing the dynamic flavor changes and the mechanisms of flavor compound formation during the processing and storage of prepared foods is highly valuable for researching flavor quality control in such products.

Modern molecular sensory science technologies reveal the flavor composition of prepared foods at the molecular level. O'Sullivan et al. (11) investigated sensory data from two cooked pork samples using gas chromatography-mass spectrometry (GC-MS) and electronic nose, finding that hexanal, 1-octen-3-ol, 2-pentylfuran, octanal, pentanal, and nonanal correlated with sensory data. Yao Wensheng et al. (12) employed headspace-gas chromatography-ion mobility spectrometry (HS-GC-IMS) to identify 2-ethylhexanol as a key flavor compound in Dezhou Braised Chicken, while ethyl acetate, 1-hexanol, 4-methyl-2-pentanone, and 1-pentanol were generated during the frying stage. Xu Min et al. (3) used Quantitative Descriptive Analysis (QDA) combined with two-dimensional gas chromatography-mass spectrometry (GC × GC-MS) to analyze the key flavor compounds and their dynamic changes during storage of Dongpo Pork. They identified key aroma compounds including 2-heptanol, 1-octen-3-ol, hexanal, (E)-2-octenal, 3-(methylthio)propanal, decanal, hexyl acetate, 2,5-dimethylpyrazine, and dimethyl trisulfide. Liu Junmei et al. (7) applied sensomics methods to identify 11 compounds, including pentanal, hexanal, (E)-2-octenal, (E)-2-undecenal, 1-octen-3-ol, and (E,E)-2,4-decadienal, as potential WOF markers in reheated braised beef.

Unlike previous studies focusing mainly on standardized meat matrices or other prepared pork dishes such as Dongpo pork and braised pork, this study focused on PSV, a traditional restaurant-style dish containing both pork and salted vegetable matrices, whose complex formulation may produce distinct storage-related volatile and taste-response patterns.

Therefore, the central question of this study was not simply whether the flavor profile of PSV changes during storage, but which instrumental markers are most associated with storage-related flavor variation in this traditional prepared meat dish. Specifically, we hypothesized that: (i) Vacuum-sealed frozen storage followed by standardized thawing and reheating would induce measurable changes in volatile and taste-related instrumental responses; (ii) ROAV and PLS-DA would help screen potential aroma-active and discriminant volatile markers associated with storage duration; and (iii) GC-IMS, E-nose, and E-tongue could detect early instrumental flavor-profile shifts before statistically significant sensory deterioration became perceptible.

## 2 Materials and methods

### 2.1 Chemicals

The selected raw materials include pork, salted vegetables, salt, cooking oil, and seasonings, all purchased from Huilin Supermarket in Chaoyang District, Beijing. The reagents used in this experiment include glacial acetic acid, chloroform, potassium iodide, starch indicator, sodium thiosulfate standard titration solution (0.1 mol/L), All chemical reagents were purchased from Sinopharm Chemical Reagent Co., Ltd. and were of analytical grade.

### 2.2 Preparation process of PSV

**Figure 1:**
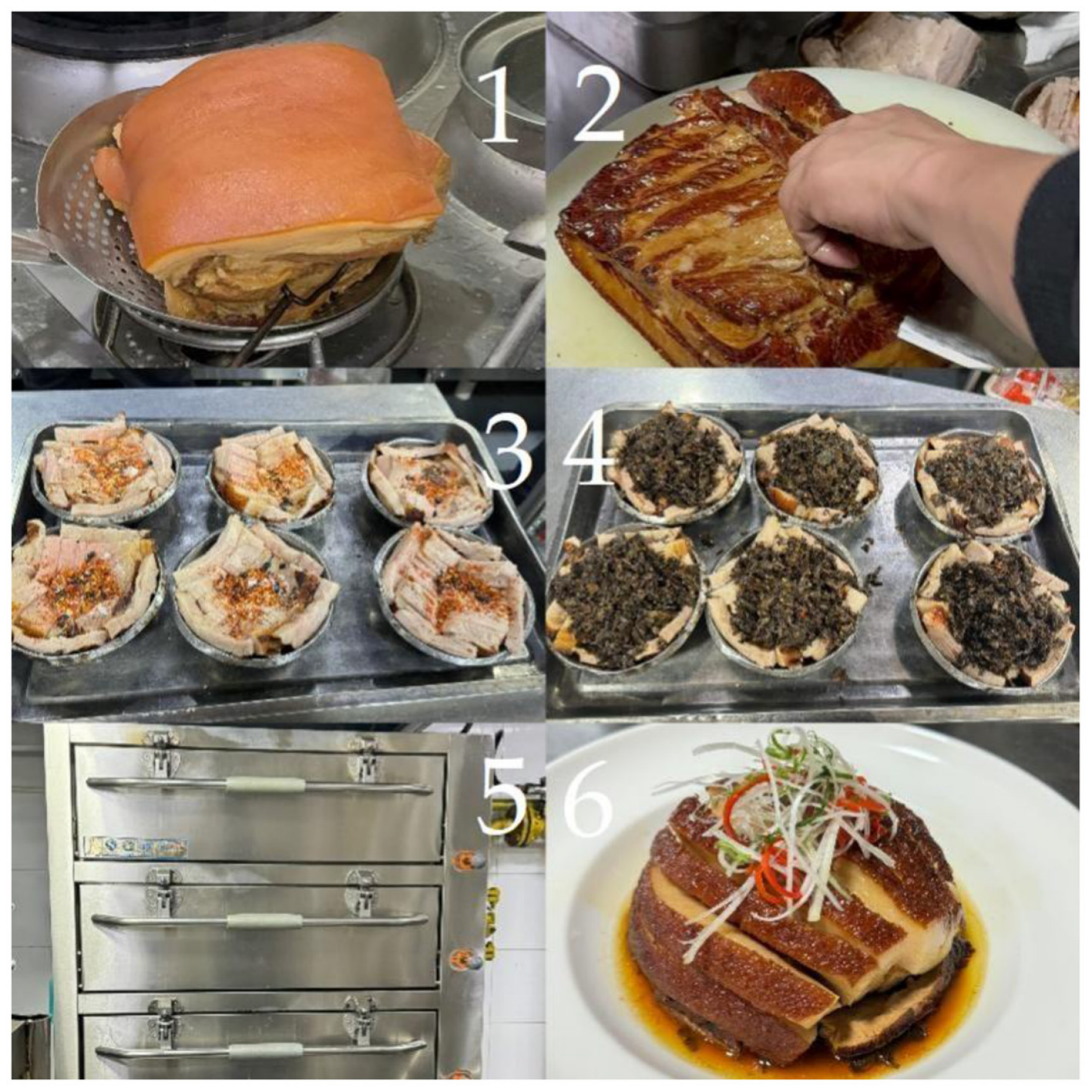
Flow chart of the production process for PSV.

All finished products of “pork with salted vegetable” were provided by Beijing Xiangyuelou Restaurant, following a Hunan cuisine recipe. The pork sample was sourced from a balanced combination of lean and adipose tissues, with a measured pH of 6.0. Fresh pork belly (750 g), with visible hair removed, was used as the raw material. The pork belly was first boiled in a sugar-colored solution for 30 min. Subsequently, the cooked pork was deep-fried in edible oil at 180 °C until the surface turned golden-brown with a blistered texture. After cooling, the pork was sliced into uniform pieces. Salted vegetables (150 g) were soaked and stir-fried prior to use. The seasoning mixture consisted of 10 g salt, 10 g monosodium glutamate, 5 g chicken essence, 5 g chili powder, 5 g fermented black beans, and 5 ml white wine. The pork and salted vegetables were arranged in a bowl and steamed at 120 °C for 90 min in a controlled steamer. After steaming, the samples were cooled to room temperature prior to further analysis (Figure 1).

After preparation, the pork with salted vegetable (PSV) was cooled to room temperature and then the samples were placed into food-grade vacuum bags, vacuum-sealed, and stored under vacuum conditions. The sealed samples were all placed in a dedicated freezer and stored frozen at −18 °C. For the fresh samples, after being completely frozen, they were thawed to room temperature and then reheated. The samples stored for one and two months were similarly thawed to room temperature prior to reheating. In each experimental group, three sample replicates were included, and the samples in different groups were prepared independently (*n* = 3 per group). Within each group, the three samples served as biological replicates for subsequent analyses. The freshly prepared sample is designated as G, the sample stored at−18 °C for 1 month as O, and the sample stored at−18 °C for 2 months as T.

Before conducting instrumental analysis, all PSV samples (including freshly prepared and stored samples) were re-heated under the same conditions. The specific method was as follows: the sealed samples were placed in a microwave oven and heated on medium heat for 5 min. Then, they were cooled to room temperature for 10 min before sampling.

### 2.3 Sensory evaluation

The sensory evaluation was conducted by a panel of 14 trained non-smoking assessors (7 males and 7 females, aged 20–30) from Beijing Union University. All panel members were habitual consumers of pork with salted vegetable and had no known food allergies. The panel members received 48 h of specialized training in descriptive analysis and scored the pork with salted vegetable at different storage times based on four items in Supplementary Table S1. Each assessor randomly received samples of pork with salted vegetable, and this process was repeated three times for each sample.

### 2.4 GC-IMS analysis

Flavor compounds were analyzed using gas chromatography-ion mobility spectrometry (GC-IMS Flavor Spec^®^) (Beijing Hanon Instrument Co., Ltd., China). First, 5.0 g of the prepared PSV was accurately weighed and placed into a 20 ml headspace vial. The sample was incubated for 20 min prior to injection, and three parallel measurements were performed for each sample. GC separation was performed using a MXT-5 column (15 m × 0.53 mm × 1.0 μm). The incubation temperature was set to 80 °C; the injection volume was 200 μL; splitless injection was used; the incubation rotation speed was 500 r/min; and the injection needle temperature was 85 °C. For gas chromatography, the column temperature was maintained at 60 °C, and high-purity nitrogen (purity ≥ 99.999%) was used as the carrier gas. The programmed pressure increase was applied as follows: an initial flow rate of 2.0 ml/min was maintained for 2 min, linearly increased to 10.0 ml/min within 8 min, and then linearly increased to 100.0 ml/min within 10 min. The chromatographic run time for the prepared PSV was 20 min, and the injection port temperature was set to 80 °C. For IMS, a tritium source (^3^H) was used as the ionization source. The migration tube length was 53 mm, and the electric field strength was 500 V/cm. The migration tube temperature was maintained at 45 °C, and high-purity nitrogen (purity ≥ 99.999%) was used as the drift gas with a flow rate of 150 ml/min. Ionization was performed in positive ion mode.

### 2.5 Identification of key volatile flavor compounds

Relative Odor Activity Value (ROAV) is a method used to analyze the contribution of each volatile component to the overall flavor of a sample (1). This method is derived from the Odor Activity Value (OAV), as shown in the following formula:


$$\begin{array}{c} OAV=\frac{C}{T}\;\\ C\%=f•C\\ ROAV_{A}=100\times \frac{OAV_{A}}{OAV_{stan}}\\ ROAV_{A}=100\times \frac{f_{stan}}{f_{A}}\times \frac{{C\%}_{A}}{{C\%}_{stan}}\times \frac{T_{stan}}{T_{A}}\approx \frac{C_{A}}{C_{stan}}\times \frac{T_{stan}}{T_{A}}\times 100 \end{array}$$

It can ultimately be simplified to:


$$\begin{array}{l} ROAV_{A}\approx \frac{C_{A}}{C_{stan}}\times \frac{T_{stan}}{T_{A}}\times 100 \end{array}$$

### 2.6 Electronic tongue analysis

Taste analysis was performed using an SA402B electronic tongue (Insent Company, Atsugi City, Japan). The electronic tongue sensing system is equipped with six sensors, namely umami (AAE), astringency (AE1), saltiness (CT0), bitterness (C00), sourness (CA0), and sweetness (GL1). First, 50 g of PSV was taken and placed in a blender, where deionized water was added for a 5-fold dilution. The mixture was then centrifuged at 4,000 rpm for 10 min. The supernatant from the sample pretreatment was filtered using qualitative filter paper to obtain the test solution. The sensors were cleaned in a cleaning solution for 90 s, followed by cleaning in a reference solution for 120 s and then another 120 s to ensure thorough cleaning. Before measurement, balance the sensors for 30 s to ensure baseline standardization. Each measurement lasts for 30 s.

The reference electrodes were Ag/AgCl electrodes supplied with the SA402B system. Filtration was performed to remove large particles and prevent sensor contamination. Because the electronic tongue mainly measures soluble taste-active compounds in the aqueous phase, the results should be interpreted as instrumental taste-response profiles rather than direct human sensory perception.

### 2.7 Electronic nose analysis

Electronic nose analysis was performed using a Gemini electronic nose (Alpha M.O.S, Toulouse, France). This electronic nose has six sensors, the response characteristics of each sensor are as follows: P10/1(combustible gases), T70/2(aromatic compounds), PA/2(organic compounds and irritant gases), P30/2(Acidic substances, alcohols), LY/G (gases with strong oxidizing properties), LY/AA(Ethanol). First, freshly cooked PSV and samples stored for 1 month and 2 months were taken, reheated, and then placed one by one into a blender. From each sample, 1 g was accurately weighed and transferred into a 10 ml headspace injection vial. Four parallels were prepared for each sample. The parameters of the electronic nose were set as follows: purified air was used as the carrier gas, the sampling time was set to 1 second, the sensor cleaning time was set to 120 s, the flow rate was set at 400 ml/min, and the analysis sampling time was set to 70 s.

### 2.8 POV analysis

The peroxide value of PSV at different storage periods was determined using the indicator titration method specified in China's food safety standard (GB 5009.227-2023). Each sample group was tested in triplicate, and the average value was calculated as the final result. Weigh 2 g to 3 g of the prepared sample (accurate to 0.001 g) into a 250 ml iodometric flask. Add 30 ml of chloroform-glacial acetic acid solution, and gently swirl to completely dissolve the sample. Accurately add 1.00 ml of saturated potassium iodide solution, stopper the flask tightly, and swirl gently for 0.5 min. Allow the mixture to stand in the dark for 3 min. Remove it, add 100 ml of water, and after shaking to mix, immediately titrate the liberated iodine with sodium thiosulfate standard titration solution (use 0.002 mol/L standard solution when the estimated peroxide value is 0.15 g/100 g or less; use 0.01 mol/L standard solution when the estimated value exceeds 0.15 g/100 g). Titrate until a pale yellow color appears, then add 1 ml of starch indicator and continue titrating with vigorous shaking until the blue color of the solution just disappears, which marks the endpoint. Conduct a blank test simultaneously.


$$\begin{array}{l} X_{1}=\frac{\left(V-V_{0}\right)\times c\times 0.1269}{m}\times 100 \end{array}$$

Where, *X*_1_ is the peroxide value, in grams per hundred grams (g/100g); V is the volume of sodium thiosulfate standard titration solution consumed by the sample, in milliliters (ml);*V*_0_ is the volume of sodium thiosulfate standard titration solution consumed by the blank test, in milliliters (ml); *c* is the concentration of the sodium thiosulfate standard titration solution, in moles per liter (mol/L); 0.1269 is the mass of iodine (g) equivalent to 1.00 ml of 1.000 mol/L sodium thiosulfate solution; *m* is the mass of the sample, in grams (g);100 is the conversion factor for expressing the result per 100 g of sample.

### 2.9 PLS-DA analysis

PLS-DA (Partial Least Squares Discriminant Analysis) is a supervised multivariate statistical method widely used for discriminant analysis. Due to its ability to effectively handle complex datasets and identify key discriminant variables, it has found extensive application in various fields such as biology, chemistry, and medicine (2). PLS-DA can be used to create correlation models linking the intensities of volatile organic compounds identified by GC-IMS to their respective sample categories.

PLS-DA analysis was performed using SIMCA 14.1 software (Umetrics, Sweden). Prior to analysis, data were mean-centered and unit variance scaled. A seven-fold cross-validation procedure was applied for internal validation. Model robustness was further assessed by 200-permutation testing. The model quality was evaluated using R2X, R2Y, and Q2 values. Variables with VIP > 1.0 were considered important discriminative markers.

### 2.10 Data analysis

For each storage group, three independent biological replicates were analyzed. Each biological replicate was measured in triplicate, and the mean value was used for statistical analysis. All the data are presented as the mean ±standard deviation. Analysis of variance (ANOVA) was used to evaluate differences among samples using SPSS software (Version 26, IBM Inc., Stamford, CA, USA); *p* < 0.05 indicated statistically significant differences using Duncan's multiple range tests. Instrumental tests were conducted in triplicate for each sample, including tests using an electronic tongue, an electronic nose, and GC-IMS (Gas Chromatography-Ion Mobility Spectrometry). GC-IMS performed qualitative analysis of target compounds by searching and matching against the built-in GC retention index (NIST 2020) database and IMS migration time database. The PCA (Principal Component Analysis) score plots and cluster analysis diagrams for the electronic tongue and electronic nose were generated using Origin 2021 (Origin Lab Corp, Northampton, MA, USA).

## 3 Results

### 3.1 Analysis of electronic nose results for PSV

**Figure 2:**
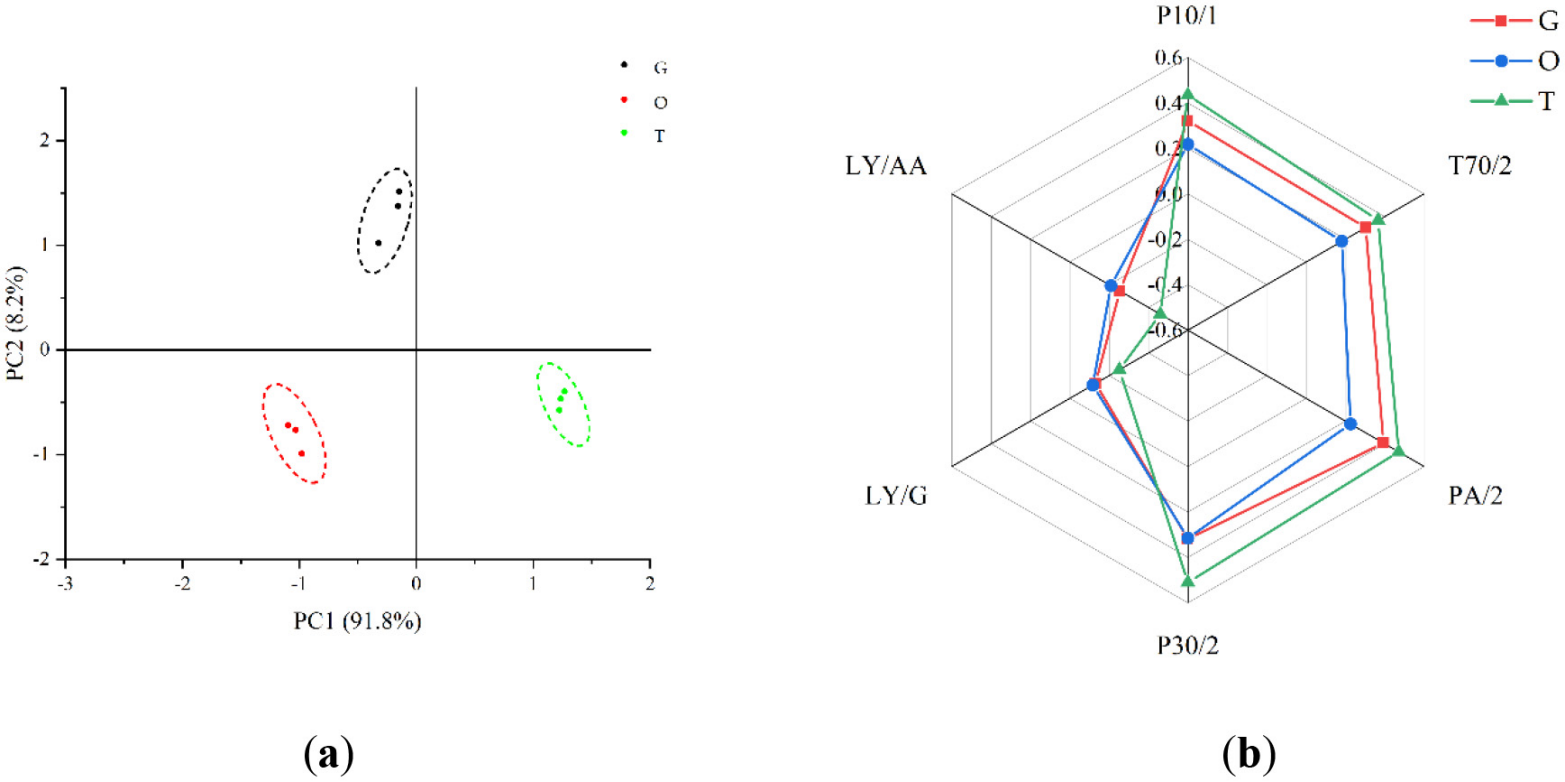
E-nose analysis. **(a)** PCA plot of the PSV in different storage periods. **(b)** Radar graph of the PSV in different storage periods. (G represents the fresh sample, O represents the sample stored for 1 month, and T represents the sample stored for 2 months).

Principal Component Analysis (PCA) is a powerful multivariate statistical method used to analyze the inherent structure of data and present it in a way that highlights similarities and differences. As shown in Figure 2a, Principal Component 1 accounts for 91.8% of the variance, and Principal Component 2 accounts for 8.2%. The smaller the distance between samples, the closer their quality characteristics. Starting from the origin of the horizontal and vertical axes, the three sample groups are located in different regions of the chart. Group G samples are located directly above, Group O samples are in the lower left region, and Group T samples are in the lower right region. The absence of significant overlap among the samples indicates that the electronic nose can distinguish PSV samples from different storage periods.

Figure 2b is a radar chart of the electronic nose sensor response values. Significant differences (*p* < 0.05) were observed among the samples for sensors P10/1, T70/2, PA/2, LY/G, and LY/AA. The change trends for sensors P10/1, T70/2, PA/2, and P30/2 were consistent across PSV samples from different storage periods, with the sensor response values first decreasing and then increasing. After 2 months of storage, the response values for sensors P10/1, T70/2, PA/2, and P30/2 in PSV samples increased compared to the freshly cooked samples. The E-nose results indicate that the volatile-response patterns differed among the G, O, and T groups. However, because E-nose sensors provide non-specific responses to broad classes of volatile compounds, these results should be interpreted as preliminary evidence of overall odor-profile variation and should be further supported by compound-level GC-IMS data. Previous studies have also suggested that compounds resulting from protein degradation or microbial decomposition may carry pungent odors, leading to an increase in the corresponding sensor readings (3).

### 3.2 Analysis of electronic tongue results for PSV

**Figure 3:**
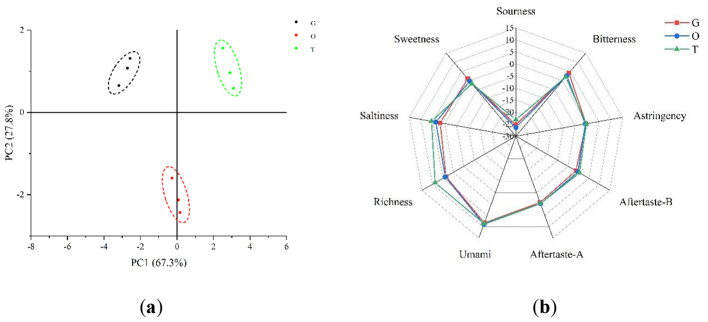
**(a)** Electronic tongue PCA data of PSV in different storage periods. **(b)** Radar graph of the PSV in different storage periods. (Note: G represents the fresh sample, O represents the sample stored for 1 month, and T represents the sample stored for 2 months).

As shown in the PCA plot of the samples in Figure 3a, Principal Component 1 accounts for 67.3% and Principal Component 2 accounts for 27.8% of the variance, which is sufficient to represent the sample information effectively. Starting from the origin of the axes, the three sample groups of prepared PSV are located in distinct regions of the chart. The absence of significant overlap among the samples indicates that the electronic tongue can differentiate PSV samples stored for different periods.

The measurement results of taste response values by the electronic tongue for PSV under different storage times are shown in Figure 3b. The samples exhibited significant differences (*p* < 0.05) in sourness, bitterness, bitter aftertaste, umami, saltiness, and sweetness, while no significant differences (*p* > 0.05) were observed in astringency, astringent aftertaste, and richness. Group G showed the highest response signal values for sourness and umami, indicating that fresh PSV showed higher electronic-tongue response values for sourness and umami sensors. As storage time increased, the signal intensity for umami sensor response generally showed a declining trend. After 1 month of storage, the richness sensor response and saltiness sensor response of the samples increased. After 2 months of storage, the bitterness sensor response, bitter aftertaste sensor response, and astringent aftertaste sensor response of the samples increased. The increase in saltiness sensor response observed at 1 month was possibly due to moisture loss from the dish. Because the electronic tongue mainly responds to water-soluble taste-active compounds, the increased bitterness response is more plausibly associated with proteolysis-derived bitter peptides and free amino acids. Volatile aldehydes and ketones may co-vary with storage-related oxidation but should not be regarded as direct contributors to the electronic tongue bitterness signal (5).

### 3.3 Sensory evaluation results

The sensory attributes of PSV were evaluated by a trained panel (*n* = 14) using a 25-point intensity scale for four parameters: flavor, color, texture, and taste. As summarized in Supplementary Table S2, the scores for all attributes declined gradually with increasing storage time. Among them, texture exhibited the largest reduction, decreasing from 19.54 ± 2.47 (G) to 18.46 ± 3.62 (T), representing a 5.5% decline. Taste showed a comparable decrease from 17.92 ± 4.59 to 17.08 ± 4.44 (−4.7%), followed by color from 20.85 ± 2.23 to 20.08 ± 2.53 (−3.7%) and flavor from 19.08 ± 3.15 to 18.69 ± 3.33 (−2.0%).

Despite these decreasing trends, one-way ANOVA with repeated measures revealed no statistically significant differences among the three storage groups for any of the four sensory attributes (*p* > 0.05). The relatively large standard deviations within each group indicated considerable individual variability among panelists, which is commonly observed in sensory evaluation even with trained assessors. This result suggests that, under the current storage conditions, the overall sensory quality of PSV remained relatively stable over the 2-month period, and the chemical changes detected by instrumental analyses (E-nose, E-tongue, and GC-IMS) had not yet reached the threshold of human perceptual discrimination.

To further explore the linear relationships between the trained panel scores and the instrumental indices, a preliminary Pearson correlation matrix was constructed based on group means (*n* = 3 storage time points, Supplementary Table S3). Owing to the limited number of storage intervals, these correlation coefficients are interpreted as exploratory trend indicators rather than confirmatory statistical inferences, and should be validated with larger datasets in future studies.

The overall sensory scores exhibited a negative correlation with peroxide value (POV, *r* = −0.999), indicating that the accumulation of primary lipid oxidation products was closely associated with the perceived non-significant decreasing sensory trends during frozen storage. Among the electronic nose sensors, PA/2 (responsive to organic compounds and pungent gases) and T70/2 (responsive to aromatic compounds) showed the strongest negative correlations with the overall sensory scores, with correlation coefficients of −0.832 and −0.790, respectively. This suggests that the increased abundance of volatile organic compounds and aromatic substances—most likely aldehydes and ketones derived from lipid oxidation—was inversely related to sensory acceptability. In contrast, the P30/2 sensor (responsive to acids and alcohols) exhibited only a weak correlation (*r* = −0.215), implying a minor role of acid/alcohol derivatives in the overall flavor chemical changes preceding statistically perceptible sensory deterioration during storage.

Regarding taste attributes, the electronic tongue umami response showed a positive correlation with the overall sensory score (*r* = 0.990), suggesting that the instrumental decrease in umami response co-varied with the non-significant decreasing trend in sensory scores. However, this relationship should be interpreted cautiously because the correlation was based on only three storage time points. The Bitterness sensor showed a moderate negative correlation (*r* = −0.532), consistent with the slight increase in bitter-related compounds (e.g., 1-octen-3-one) observed in the GC-IMS profiles at the 2-month storage point. However, the Richness sensor exhibited the weakest association with sensory scores (*r* = −0.120), possibly due to the anomalously high response recorded in the 1-month stored samples.

Collectively, these trend-based correlation results suggest that aldehydes, ketones, and umami-related taste responses may be associated with early instrumental flavor-profile shifts in PSV during frozen storage and reheating. Nevertheless, given the exploratory nature of this analysis, future studies incorporating a larger number of storage time points and a full Partial Least Squares Regression (PLSR) model are warranted to establish quantitative predictive relationships between instrumental markers and human sensory perception.

### 3.4 POV analysis

**Figure 4:**
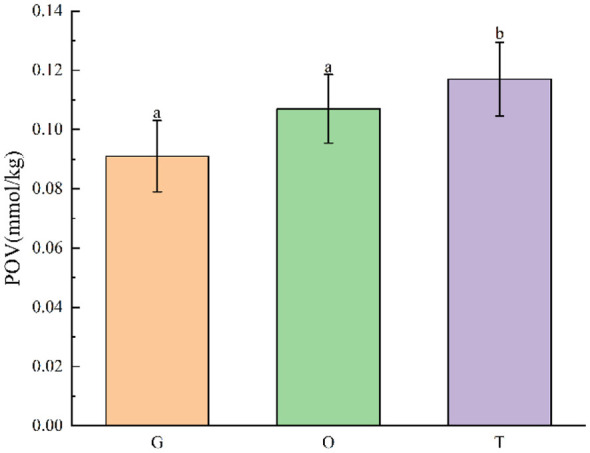
Peroxide Value Results of PSV (Note: G represents the fresh sample, O represents the sample stored for 1 month, and T represents the sample stored for 2 months).

To further validate the degree of lipid rancidity in PSV, the peroxide value—a key indicator for assessing lipid oxidation (6, 7)—was measured across different storage periods. The POV increased from 0.09 ± 0.02 g/100 g in G to 0.11 ± 0.02 g/100 g in O and 0.12 ± 0.02 g/100 g in T. As shown in Figure 4, the peroxide value of PSV gradually increased with extended storage time, suggesting a tendency toward increased primary lipid oxidation products. These hydroperoxides may subsequently decompose during thawing and reheating to generate secondary volatile compounds. This result is consistent with the findings obtained from GC-IMS analysis.

### 3.5 Fingerprint analysis

**Figure 5:**
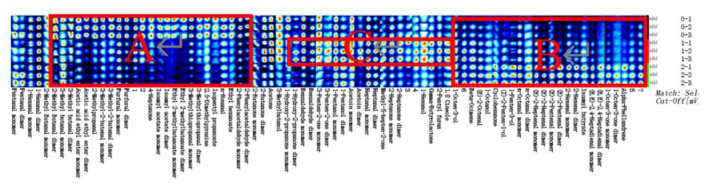
Fingerprint spectra of volatile compounds in PSV at different storage times. (Note: The numbers represent compounds which could not be accurately identified, 0 represents the samples of Group G, 1 represents the samples of Group O, and 2 represents the samples of Group T).

To further investigate the differences in volatile flavor compounds of PSV during different storage periods, fingerprint spectra (Gallery Plot) were generated using a plugin (Figure 5). Figure 5 illustrates the complete volatile organic compound profiles of each sample and the differences in volatile organic compounds among the samples. Darker red indicates higher concentrations, while lighter colors indicate lower concentrations (13).

The compounds in Area A of the figure exhibited the highest concentrations in the freshly cooked PSV, mainly including: 2-phenylethanal, n-nonanal, 3-(methylthio)propanal, 2-methylpropanal, 3-methylbutanal, 2-methylbutanal, 3-methyl-2-butenal, ethyl hexanoate, isoamyl propionate, ethyl 2-methylbutyrate, isoamyl acetate, ethyl acetate, 2,5-dimethylpyrazine, furfural, and 4-heptanone. The compounds in Area C showed the highest concentrations in PSV after 1 month of storage, mainly including: 1,8-cineole, 2-pentylfuran, γ-butyrolactone, 1-hexanol, 2-heptanone, 5-hepten-2-one methyl, heptanal, acetoin (3-hydroxy-2-butanone), 1-pentanol, 3-penten-2-one, benzaldehyde, and 1-hydroxy-2-propanone. The compounds in Area B displayed the highest concentrations in PSV after 2 months of storage, mainly including: α-phellandrene, 1-octen-3-one, (E,E)-2,4-heptadienal, isoamyl butyrate, 2-hexenal, (E)-2-pentenal, (E)-2-heptenal, n-octanal, 1-penten-3-ol, (Z)-2-penten-1-ol, cyclohexanone, 1-octanol, (E)-2-octenal, β-ocimene, and 1-octen-3-ol.

The results from the fingerprint spectra indicate that the main volatile components in Group G samples were primarily aldehydes and esters. The main volatile components in Group O samples were predominantly ketones, while those in Group T samples were mainly aldehydes, ketones, and esters.

### 3.6 Qualitative results of volatile compounds

A total of 79 flavor compounds were identified by GC-IMS, including 33 aldehydes, 8 alcohols, 16 ketones, 10 esters, 1 pyrazine, 1 furan, 3 terpenes, and 7 unidentified compounds. The results of GC-IMS were shown in Supplementary Table S4.

**Figure 6:**
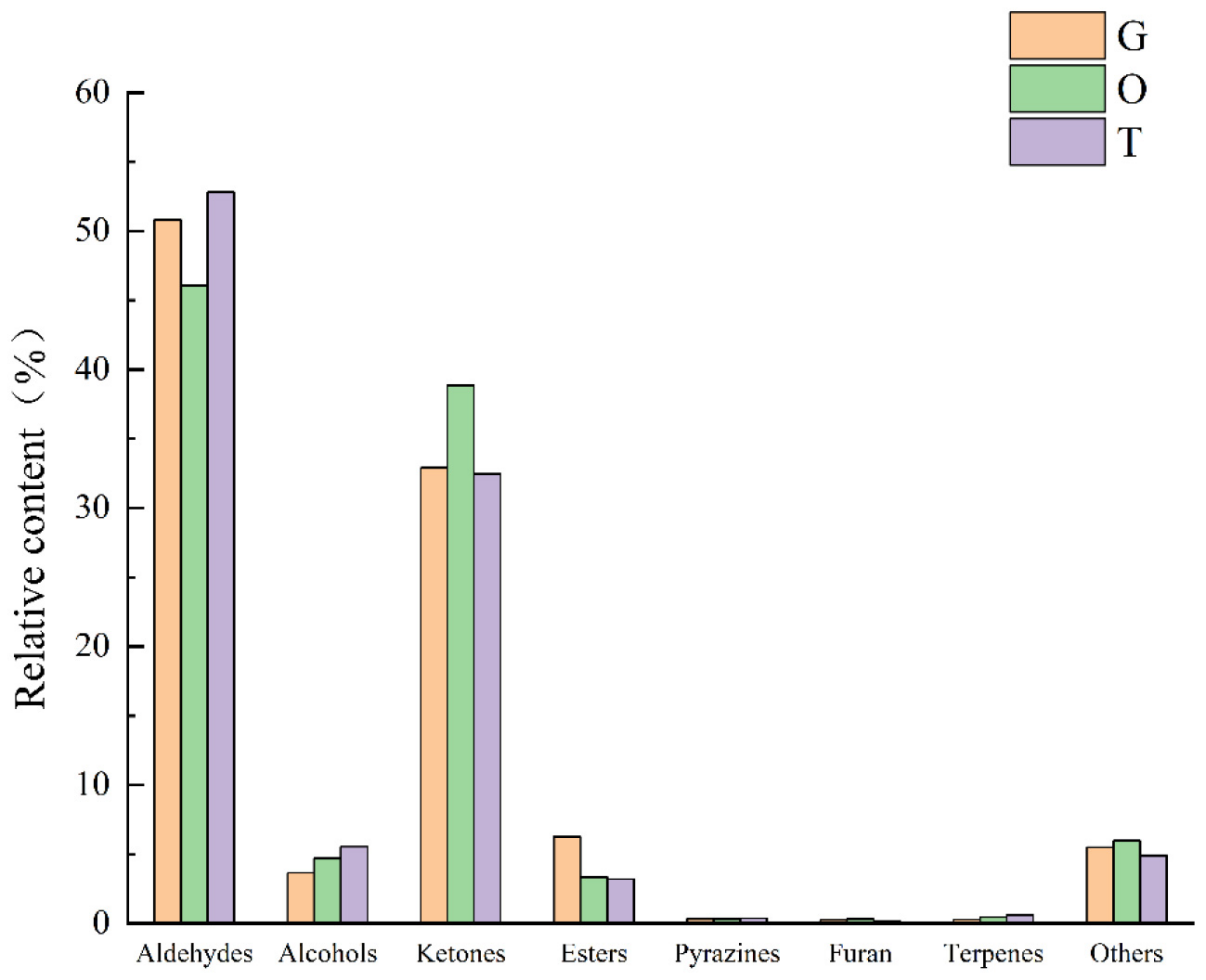
Relative Content of Volatile Compounds in PSV. (Note: G represents the fresh sample, O represents the sample stored for 1 month, and T represents the sample stored for 2 months).

Among all samples, the proportions of each type of compound relative to the total volatile compounds were: aldehydes 46.05%−52.80%; ketones 32.44%−38.86%; esters 3.21%−6.26%; alcohols 3.66%−5.54%; terpenes 0.29%−0.61%; pyrazines 0.32%−0.35%; furans 0.16%−0.32%. The most abundant flavor compounds in PSV were primarily aldehydes, ketones, and esters. The relative content of volatile compounds in PSV is shown in Figure 6.

Aldehydes are significant contributors to the flavor of meat products, primarily generated through lipid oxidation or Strecker degradation in the Maillard reaction (3, 14). In the freshly cooked samples (G), 3-methylbutanal (7.49%), 2-methylbutanal (5.5%), and benzaldehyde (3.27%) accounted for relatively high proportions. 3-Methylbutanal and 2-methylbutanal are produced via Strecker degradation reactions (15), imparting malt, cocoa, and fatty aromas (7). Benzaldehyde, likely derived from the decomposition of phenylalanine or linoleic acid, presents a bitter almond flavor (16). In contrast, in the T group samples, the proportions of (E)-2-heptenal, (E)-2-pentenal, pentanal, and heptanal increased. Literature indicates these aldehydes are also key flavor factors in PSV (5), strongly associated with fat oxidation, and are one of the main causes of warmed-over flavor (WOF). Aldehydes had the highest proportion in the T group samples (52.80%), followed by the G group (50.8%), and the lowest relative content in the O group (46.05%). This may be due to the increasing degree of lipid oxidation over time, gradually raising the relative content of aldehydes.

Ketones likely originate mainly from lipid oxidation, the Maillard reaction, and microbial esterification. These compounds possess distinctive flavors and significantly impact meat product flavor. They are products of further oxidation of aldehydes (3) and, with increasing storage time, can substantially affect the flavor and sensory characteristics of meat products. 1-Octen-3-one has a relatively high content and low threshold, contributing significantly to the overall flavor with a strong mushroom and metallic odor (17, 18) (14, 15). 3-Hydroxy-2-butanone accounted for a relatively high proportion in all groups (4.37%, 6.11%, 4.75%). It originates from glucose catabolism or oleic acid oxidation and exhibits fatty odor characteristics (19). Ketones had the highest proportion in the O group (38.86%), followed by the G group (32.90%), and the lowest relative content in the T group (32.44%). The lower relative proportion of ketones in the T group, despite the higher aldehyde proportion, may reflect the different formation and depletion kinetics of volatile compound classes. Ketones may accumulate transiently during the intermediate storage stage, as observed in the O group, but may subsequently decrease because of volatilization during thawing and reheating, further oxidation, reduction to corresponding alcohols, or participation in secondary reactions. In addition, the values reported here represent relative proportions of compound classes rather than absolute concentrations. Thus, the marked increase in aldehydes in the T group may proportionally dilute the relative contribution of ketones. This result therefore does not necessarily contradict the occurrence of lipid oxidation, but instead suggests that aldehydes were the dominant secondary oxidation-related volatiles after prolonged frozen storage and reheating.

Alcohol compounds generally derive from the oxidation of unsaturated fatty acids during meat processing and play an important role in the overall flavor formation and richness of dishes (20–22). 1-Octen-3-ol and 1-pentanol accounted for relatively high proportions in all samples. 1-Octen-3-ol, originating from lipid oxidation, imparts a mushroom flavor to meat products (18, 19). 1-Pentanol may arise from linoleic acid oxidation during cooking, offering fruity and oily flavors (23). Alcohols had the highest proportion in the T group samples (5.54%), followed by the O group (4.69%), and the lowest relative content in the G group (3.66%).

Ester compounds primarily originate from esterification reactions and lipid oxidation (24), possessing sweet and typical fruity aromas (25). Ethyl acetate accounted for a relatively high proportion in all samples and is a representative ester compound in PSV, emitting subtle grape and cherry-like aromas (26). The relative content of esters gradually decreased from the G group (6.26%) to the O group (3.34%) and the T group (3.21%) with increasing storage time, likely due to gradual loss during reheating and storage. This trend is consistent with the analysis reported in the literature by Siyang Deng et al. (27).

Terpenes are volatile compounds with low odor thresholds, with the general formula (C5H8) n, where n represents the number of isoprene units. They are typically considered plant metabolites (28). Terpenes generally provide herbal, floral, and fruity aromas. This study mainly detected three terpenes: α-phellandrene, ocimene, and eucalyptol. Their content changes were not significant (0.29%−0.61%). α-Phellandrene provides a note of earthiness and fennel. Ocimene offers floral, woody, and fruity aromas. Eucalyptol imparts camphoraceous and cooling herbal notes.

Pyrazines and furans are common products of reactions involving free amino acids, peptides, and proteins under thermal treatment (12, 29). This study detected 2,5-dimethylpyrazine and 2-pentylfuran in all samples. 2,5-Dimethylpyrazine can impart cocoa and coffee flavors to meat products (30). Its content was highest in the T group (0.35%), followed by the G group (0.34%), and lowest in the O group (0.32%). Furans may originate from the Maillard reaction. 2-Pentylfuran is a non-carboxylic acid compound derived from linoleic acid and other fatty acids, possessing nutty and vegetable-like flavors (31). Its content was highest in the O group (0.32%), followed by the G group (0.26%), and lowest in the T group (0.16%).

The changes in Maillard- and Strecker-derived compounds should also be interpreted cautiously. Compounds such as 3-methylbutanal, 2-methylbutanal, 2-phenylacetaldehyde, furfural, and 2,5-dimethylpyrazine are generally associated with thermal reactions during boiling, frying, steaming, and reheating rather than with active Maillard reaction during frozen storage itself. Under −18 °C frozen storage, the formation of new Maillard products is expected to be limited. Therefore, the observed changes in these compounds more likely reflect volatilization, oxidative degradation, matrix binding or release, and redistribution during thawing and reheating. This interpretation helps distinguish compounds associated with the original cooked aroma from those more closely related to storage- and reheating-associated volatile shifts.

Focusing specifically on the statistically significant differences shown in Supplementary Table S1, group-specific enrichment patterns could be identified when compounds with significantly higher relative contents in one group than in the other two groups were considered (*p* < 0.05). In the G group, 23 volatile compounds/signals were significantly enriched, including 2-phenylacetaldehyde (monomer and dimer), ethyl hexanoate, acetic acid ethyl ester (monomer and dimer), 2-methylpropanal, 2-methylbutanal dimer, 3-methylbutanal dimer, 3-penten-2-one monomer, 3-methyl-2-butenal (monomer and dimer), furfural (monomer and dimer), unidentified compound 2, isoamyl acetate (monomer and dimer), 3-methylthiopropanal (monomer and dimer), 4-heptanone, ethyl 2-methylbutanoate (monomer and dimer), 2,5-dimethylpyrazine, and isopentyl propanoate. In the O group, 11 compounds/signals were significantly enriched, including 1,8-cineole, 2-pentylfuran, acetone, acetoin dimer, 1-hydroxy-2-propanone dimer, 3-penten-2-one dimer, 2-heptanone (monomer and dimer), unidentified compound 4, 1-hexanol, and methyl-5-hepten-2-one. In the T group, 22 compounds/signals were significantly enriched, including (E)-2-octenal, 1-octanol, (E,E)-2,4-heptadienal (monomer and dimer), n-octanal (monomer and dimer), (E)-2-heptenal (monomer and dimer), 1-octen-3-one (monomer and dimer), 3-methylbutanal monomer, (E)-2-pentenal (monomer and dimer), (Z)-2-penten-1-ol, 1-penten-3-ol, 2-hexenal (monomer and dimer), cyclohexanone, unidentified compounds 5 and 7, α-phellandrene, and isoamyl butyrate.

Overall, these significance-focused results further support the storage-dependent redistribution of volatile compounds in PSV. Fresh samples (G) were characterized by significantly higher levels of Strecker/Maillard-related aldehydes, furans/pyrazines, and fruity esters, which may be associated with fresh cooked, roasted, and fruity aroma notes. After 1 month of frozen storage (O), significantly enriched compounds were mainly ketones, furan, alcohol, and terpene-related signals, suggesting the accumulation of intermediate oxidation products. After 2 months of storage (T), a series of lipid-oxidation-related aldehydes and alcohols, especially unsaturated aldehydes and 1-octen-3-one, increased significantly, indicating that prolonged storage promoted the formation of oxidation-derived volatiles and potential warmed-over flavor markers.

### 3.7 ROAV analysis

The odor characteristics of volatile compounds do not depend solely on their concentration but rather on their odor thresholds and overall content. These factors determine the contribution of volatile compounds to the overall flavor profile (31). Therefore, the Relative Odor Activity Value (ROAV) can be used to assess the contribution of volatile compounds to the overall aroma characteristics. A higher ROAV indicates a greater contribution of that compound to the overall flavor. Compounds with ROAV ≥ 1 are considered candidate aroma-active compounds of the sample, while those with 0.1 ≤ ROAV < 1 are auxiliary flavor compounds that modify the overall sample flavor (31). In this experiment, ROAV values for each flavor compound were calculated using 1-octen-3-one as the reference (ROAV = 100). Because it showed a high relative abundance and has a low odor threshold. The ROAV calculation results were shown in Supplementary Table S5.

**Figure 7:**
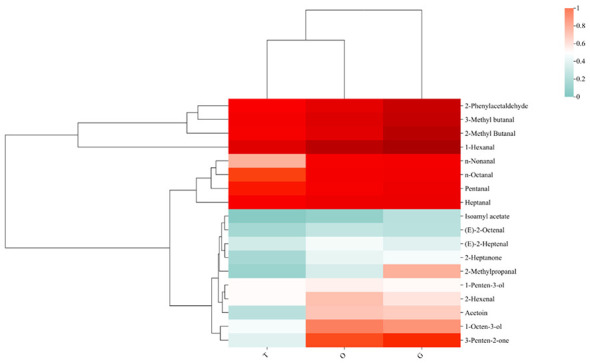
Heatmap of ROAV values for key and important flavor compounds in PSV. (Note: G represents the fresh sample, O represents the sample stored for 1 month, and T represents the sample stored for 2 months).

Figure 7 is a heatmap showing the ROAV values of the key and important flavor compounds in the samples. In Group G (freshly cooked), 10 candidate aroma-active compounds (ROAV ≥ 1) and 9 auxiliary flavor compounds (0.1 ≤ ROAV < 1) were detected. The 10 candidate aroma-active compounds were pentanal, n-octanal, n-nonanal, heptanal, 3-methylbutanal, 2-phenylethanal, 2-methylbutanal, 1-octen-3-one, hexanal, and 3-penten-2-one. The 9 auxiliary flavor compounds were acetoin, 2-methylpropanal, 2-hexenal, 2-heptanone, 1-penten-3-ol, 1-octen-3-ol, **(E)**-2-octenal, **(E)**-2-heptenal, and isoamyl acetate. These substances together constitute the basic aroma profile of PSV, presenting green, fruity, cocoa, and distinct fatty notes.

It can be observed that with increasing storage time, the number and types of candidate aroma-active compounds and auxiliary flavor compounds in Group O (one-month storage) did not change significantly, and their ROAV values showed little variation. However, after 2 months of storage (Group T), the ROAV values of 3-penten-2-one and n-nonanal decreased from 2.51 and 1.29 to 0.79 and 0.3, respectively. Furthermore, the ROAV values of other candidate aroma-active compounds decreased further. This reduction suggests a change in the relative aroma-contribution pattern during prolonged storage and may be associated with lipid oxidation-related volatile redistribution. However, because ROAV values were derived from GC-IMS relative signal intensities, these results should not be interpreted as direct evidence of stronger sensory off-flavor. This also demonstrates that the characteristic flavor of PSV undergoes instrumental flavor-profile shifts during storage. The decrease in ROAV values indicates a shift in the relative aroma-contribution pattern rather than a direct decrease in absolute concentration. Because ROAV values were calculated from GC-IMS relative signal intensities, these results should be used to screen candidate odor contributors rather than to confirm sensory flavor loss.

### 3.8 Multivariate statistical analysis

**Figure 8:**
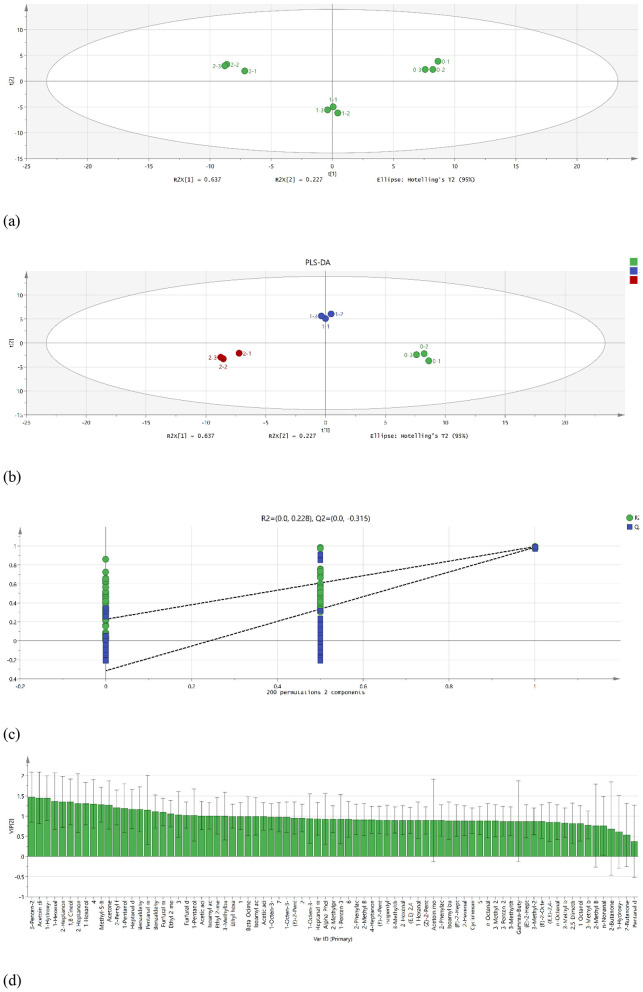
PLS-DA results: **(a)** PCA score plot; **(b)** PLS-DA score plot; **(c)** PLS-DA validation plot; **(d)** VIP score plot. (Note:0 represents the samples of Group G, 1 represents the samples of Group O, and 2 represents the samples of Group T).

Principal component analysis (PCA) was initially conducted on the data, and the results are shown in Figure 8a. Distinct differences were observed among the samples, indicating the suitability of proceeding with PLS-DA modeling. As can be inferred from the figure, PC1 (63.7%) and PC2 (22.7%) together account for 86.4% of the total variance. Group G (Sample 0) is located on the right side of the PCA plot, Group O (Sample 1) at the bottom, and Group T (Sample 2) on the left side. This indicates significant differences among the groups, suggesting that the volatile components of PSV undergo marked changes during different storage stages. Collectively, principal component analysis successfully enables the identification and discrimination of the volatile flavor components of pork at various processing stages.

In the PLS-DA score plot (Figure 8b), the first principal component and the second component explained 63.7% and 22.7% of the overall variance, respectively. The PLS-DA score plot directly represents the similarity and differences among samples. The greater the difference between two samples, the farther apart their relative positions are on the score plot; conversely, samples with similar characteristics are positioned closer together (32). The freshly cooked samples (G) were located on the right side of the plot, the 1-month stored samples (O) in the middle, and the 2-month stored samples (T) on the left side.

As shown in the PLS-DA score plot (Figure 8c), the samples were clearly separated into three distinct groups, indicating significant differences in volatile compounds among PSV samples stored for different periods. A permutation test with 200 iterations was performed for cross-validated predictive variance analysis to evaluate the model indices. The independent variable fitting index R2X was 0.864, and the dependent variable fitting index R2Y was 0.988. R2X and R2Y represent the percentage of information in the X and Y matrices, respectively, that can be explained by the model. A difference of R2X–R2Y < 0.3 indicates the model is relatively reliable. The Q2 value of 0.975, close to 1, suggests the model fits reasonably well. To prevent overfitting, a 200-permutation test was conducted, yielding Figure 4c. The Q2 regression line intersected the vertical axis below zero, and both the R2 (0.228) and Q2 (-0.315) values were less than the threshold of 1.0. This confirms the model is not overfitted and is reliable (33). These results can be used to analyze PSV samples from different storage periods.

The VIP scores are shown in Figure 8d. The Variable Importance in Projection (VIP) score is a method within the PLS-DA model to evaluate the relative contribution of each variable to the classification and differentiation of sample groups. A higher VIP value indicates a greater differentiation of the aroma compound among groups, meaning this flavor compound is more important for the discriminant classification of the overall dish flavor. A VIP > 1 indicates that the compound is a key discriminant marker in the PLS-DA model, contributing significantly to the overall flavor of the dish (34). A total of 19 compounds with VIP > 1 were screened out, as shown in Figure 8c. Nineteen variables with VIP > 1 were identified and are listed in Supplementary Table S6. These included 5 ketones, 5 aldehydes, 4 esters, 4 alcohols, 1 terpene, and 1 heterocyclic compound. Among them, the 3-penten-2-one dimer, acetoacetic acid dimer, and 1-hydroxy-2-propanone dimer each exhibited VIP scores exceeding 1.4, serving as the primary discriminatory compounds. The dimer signals observed in GC-IMS were interpreted cautiously because such signals may arise from concentration-dependent ion clustering. Therefore, monomer signals and compounds with consistent storage trends were prioritized in the discussion.

## 4 Discussion and conclusions

### 4.1 Strengths and limitations

In contrast to most previous studies on flavor changes in meat products, which have predominantly concentrated on industrially processed or standardized matrices, the present study investigated a dish prepared under traditional restaurant-style conditions, where the processing steps are more intricate, ingredient compositions are more variable, and operational parameters are less uniform. Consequently, the flavor evolution patterns obtained under such realistic culinary conditions offer more practical guidance for actual catering scenarios. The study employed a trained sensory panel for scoring and combined these scores with instrumental data from electronic nose, electronic tongue, and GC-IMS, followed by exploratory correlation analyses. The results revealed that the overall sensory scores exhibited a near-perfect negative correlation with peroxide value (POV) (*r* = −0.999) and a near-perfect positive correlation with umami response from the electronic tongue (*r* = 0.990). Additionally, the responses of sensors in the electronic nose sensitive to organic compounds and aromatic compounds were also significantly negatively correlated with sensory scores. Although these correlations are constrained by the limited number of storage time points, they collectively support the conclusion that aldehydes, ketones, and umami-related compounds are the primary drivers of flavor variation in PSV during storage, and provide a feasible reference for catering operations to optimize storage duration and maintain flavor quality.

Several limitations of this study should be acknowledged. First, due to the limited number of storage time points, the correlation analyses remain exploratory and the results should be interpreted with caution. Second, only peroxide value (POV) was employed as an indicator of lipid oxidation, which primarily reflects primary oxidation products and is insufficient to fully elucidate the oxidation mechanisms. The increase in POV should be interpreted as evidence for the accumulation of primary lipid oxidation products rather than as a direct measure of secondary volatile compounds. During frozen storage, lipid oxidation is slowed but not completely prevented, especially in a complex cooked meat matrix containing residual oxygen and unsaturated fatty acids. Hydroperoxides formed from lipid oxidation may remain relatively unstable and can decompose during thawing and reheating, generating secondary volatile compounds such as saturated aldehydes, unsaturated aldehydes, ketones, alcohols, and furans. Therefore, the gradual increase in POV from G to T is chemically consistent with the higher relative abundance of several storage-related aldehydes, including hexanal, heptanal, n-octanal, n-nonanal, (E)-2-heptenal, and (E)-2-octenal. However, because POV mainly reflects primary oxidation products, it should not be regarded as direct quantitative evidence for the formation of these secondary volatiles. Future studies are warranted to incorporate additional indicators, including thiobarbituric acid reactive substances (TBARS), acid value, free fatty acids, pH, water activity, and quantification of hexanal, in order to more systematically reveal the chemical pathways and oxidative processes underlying flavor deterioration. Third, although the PLS-DA model was validated using seven-fold cross-validation and 200 permutation tests, the limited sample size precludes confirmatory inference; therefore, the PLS-DA results should be regarded as exploratory rather than confirmatory. Fourth, because GC-IMS provides relative signal intensities rather than absolute concentrations, the ROAV values calculated herein are semi-quantitative in nature; they served primarily to prioritize compounds for discussion rather than to assign definitive aroma activity. Fifth, it should be noted that the storage durations employed in this study are not intended to represent actual storage practices in real-world catering scenarios. Rather, these durations were chosen to amplify the flavor changes in the dish for the purpose of academic investigation, and do not aim to determine the true shelf life of the product. The extended storage periods were used solely to facilitate the detection of volatile compositional shifts and are not recommended or representative of typical consumption or storage conditions. Sixth, although the reheating procedure was consistently applied across all sample groups to eliminate differential treatment effects, the reheating step itself may induce additional volatile formation (e.g., lipid oxidation and Strecker degradation products) that could influence the observed profiles. Future studies incorporating non-reheated fresh controls would be valuable to further dissect the contributions of storage versus reheating to flavor changes. Seventh, the present study focused primarily on instrumental and sensory flavor quality; microbiological indicators, such as total viable counts or specific spoilage organisms, were not monitored. While the samples were stored under frozen conditions (−18 °C) to minimize microbial risks, future studies examining actual shelf-life safety should include comprehensive microbiological assessments. Eighth, although the samples were vacuum-sealed to minimize oxygen exposure, other packaging-related parameters (e.g., precise headspace ratio, residual oxygen level, and potential temperature fluctuations during freezer storage) were not exhaustively controlled in this study, which may contribute to batch-to-batch variability. Future work with more tightly controlled packaging conditions is recommended.

## 5 Conclusions

This study integrated GC–IMS, electronic nose, electronic tongue, POV analysis, and sensory evaluation to investigate instrumental and sensory changes in pork with salted vegetable (PSV) during vacuum-sealed frozen storage followed by standardized thawing and reheating. A total of 79 volatile compounds were identified by GC–IMS, including 33 aldehydes, 8 alcohols, 16 ketones, 10 esters, 1 pyrazine, 1 furan, 3 terpenes, and 7 unidentified compounds. Aldehydes, ketones, esters, and alcohols were the dominant volatile classes contributing to the instrumental differentiation among storage stages. The instrumental results indicated storage-related shifts in volatile and taste-response profiles. Aldehydes showed the highest relative proportion in the 2-month group, whereas ketones reached the highest relative proportion in the 1-month group, suggesting that different volatile classes may follow distinct formation and depletion patterns during frozen storage, thawing, and reheating. POV increased slightly with storage time, indicating a tendency toward accumulation of primary lipid oxidation products. However, because POV does not directly quantify secondary volatile compounds, lipid oxidation-related interpretations should remain cautious. ROAV analysis identified several candidate aroma-active compounds, including pentanal, hexanal, heptanal, n-octanal, n-nonanal, 3-methylbutanal, 2-methylbutanal, 2-phenylethanal, 1-octen-3-one, and 3-penten-2-one. Because ROAV values were calculated from GC–IMS relative signal intensities rather than absolute concentrations, these compounds should be regarded as potential odor contributors rather than definitive key aroma compounds. PLS-DA further screened 19 discriminant volatile markers, mainly aldehydes, ketones, alcohols, and esters, but these markers should be interpreted as exploratory indicators of group separation rather than direct evidence of sensory deterioration. Sensory evaluation showed slight decreasing trends in flavor, color, texture, and taste scores during storage, but no statistically significant differences were observed among the three storage groups. Therefore, the chemical and instrumental changes detected in this study had not yet reached the threshold of statistically significant human sensory discrimination under the present experimental conditions. Overall, the results suggest that GC–IMS combined with electronic nose and electronic tongue analyses can detect early instrumental flavor-profile shifts in PSV during frozen storage and reheating, while sensory quality remained relatively stable over the two-month period. Future studies should include more storage intervals, larger sensory datasets, absolute quantification of key volatiles, and additional oxidation and proteolysis indicators to further clarify the relationship between instrumental markers and sensory perception.

## Data Availability

The raw data supporting the conclusions of this article will be made available by the authors, without undue reservation.
