# Supplementary material for: Evolution of instrumental flavor profiles in pork with salted vegetable during frozen storage revealed by GC-IMS, E-nose, and E-tongue
Source: Front Nutr. 2026 Jul 27;13:1880506. doi: 10.3389/fnut.2026.1880506 (PMC13455502; doi:10.3389/fnut.2026.1880506)
Supplement: Supplementary file 1 [file Data_Sheet_1.zip › 梅菜扣肉补充材料.docx]

**Table S1.** Sensory scale of pork with salted vegetable.

| Item | Standard | Score (points) |
| --- | --- | --- |
| Flavor (25 points) | Strong aroma of pork with salted vegetable | 20-25 |
|  | Relatively strong aroma of pork with salted vegetable | 10-19 |
|  | Faint aroma of pork with salted vegetable | 1-9 |
| Color (25 points) | The pork skin of pork with salted vegetable is bright red, with a sauce-red color | 20-25 |
|  | The pork skin of pork with salted vegetable is slightly bright red, with a slightly sauce-red color | 10-19 |
|  | The color of pork with salted vegetable is too dark or too light, with no luster | 1-9 |
| Texture (25 points) | The pork with salted vegetable is moderately tender, with a rich texture | 20-25 |
|  | The lean meat of pork with salted vegetable is overly tender, while the fatty meat is greasy | 10-19 |
|  | The lean meat of pork with salted vegetable is dry and hard, while the fatty meat is greasy | 1-9 |
| Taste (25 points) | The pork with salted vegetable is fragrant and rich, with a non-greasy taste | 20-25 |
|  | The pork with salted vegetable is relatively fragrant but slightly greasy upon ingestion | 10-19 |
|  | The pork with salted vegetable has a bland taste upon ingestion | 1-9 |

**Table S2.** Sensory Scores of pork with salted vegetable.

|  | **Flavor** | **Color** | **Texture** | **Taste** |
| --- | --- | --- | --- | --- |
| **G (Fresh)** | 19.08 ± 3.15 | 20.85 ± 2.23 | 19.54 ± 2.47 | 17.92 ± 4.59 |
| **O (1 month)** | 18.69 ± 3.33 | 20.62 ± 2.72 | 18.62 ± 3.78 | 17.46 ± 4.82 |
| **T (2 months)** | 18.69 ± 3.33 | 20.08 ± 2.53 | 18.46 ± 3.62 | 17.08 ± 4.44 |

Note: Sensory attributes were evaluated by a trained panel (n=14) on a 25-point intensity scale (0 = none, 25 = extremely strong).

**Table S3.** Exploratory Pearson Correlation Matrix between Sensory Scores, POV, E‑nose, and E‑tongue Responses.

| Variable | Overall Sensory Score | POV | P10/1 | T70/2 | PA/2 | P30/2 | LY/G | LY/AA | Sourness | Bitterness | Astringency | Umami | Richness | Saltiness | Sweetness |
| --- | --- | --- | --- | --- | --- | --- | --- | --- | --- | --- | --- | --- | --- | --- | --- |
| Overall Sensory Score | 1.000 |  |  |  |  |  |  |  |  |  |  |  |  |  |  |
| POV | −0.999 | 1.000 |  |  |  |  |  |  |  |  |  |  |  |  |  |
| P10/1 | −0.686 | 0.686 | 1.000 |  |  |  |  |  |  |  |  |  |  |  |  |
| T70/2 | −0.790 | 0.819 | 0.979 | 1.000 |  |  |  |  |  |  |  |  |  |  |  |
| PA/2 | −0.832 | 0.851 | 0.987 | 0.998 | 1.000 |  |  |  |  |  |  |  |  |  |  |
| P30/2 | −0.215 | 0.241 | 0.778 | 0.849 | 0.822 | 1.000 |  |  |  |  |  |  |  |  |  |
| LY/G | 0.349 | −0.367 | −0.932 | −0.951 | −0.940 | −0.899 | 1.000 |  |  |  |  |  |  |  |  |
| LY/AA | 0.405 | −0.423 | −0.947 | −0.974 | −0.965 | −0.934 | 0.993 | 1.000 |  |  |  |  |  |  |  |
| Sourness | −0.477 | 0.486 | 0.822 | 0.802 | 0.813 | 0.296 | −0.519 | −0.519 | 1.000 |  |  |  |  |  |  |
| Bitterness | −0.532 | 0.512 | 0.583 | 0.553 | 0.574 | 0.066 | −0.241 | −0.255 | 0.910 | 1.000 |  |  |  |  |  |
| Astringency | −0.825 | 0.823 | 0.913 | 0.917 | 0.918 | 0.549 | −0.708 | −0.722 | 0.861 | 0.721 | 1.000 |  |  |  |  |
| Umami | 0.990 | −0.987 | −0.624 | −0.729 | −0.773 | −0.126 | 0.358 | 0.429 | −0.397 | −0.436 | −0.775 | 1.000 |  |  |  |
| Richness | −0.120 | 0.130 | 0.438 | 0.477 | 0.480 | 0.889 | −0.607 | −0.666 | 0.024 | −0.152 | 0.247 | 0.063 | 1.000 |  |  |
| Saltiness | 0.352 | −0.367 | −0.881 | −0.917 | −0.930 | −0.695 | 0.710 | 0.769 | −0.728 | −0.582 | −0.802 | 0.466 | −0.345 | 1.000 |  |
| Sweetness | −0.298 | 0.278 | −0.450 | −0.520 | −0.522 | −0.821 | 0.626 | 0.622 | −0.214 | −0.218 | −0.450 | −0.365 | −0.842 | 0.424 | 1.000 |

Note: Correlation coefficients (r) were calculated based on group means of three storage time points (n = 3: G, O, T). Bold values indicate strong correlations (|r| ≥ 0.8) discussed in the main text. Owing to the limited number of storage intervals, this matrix is presented as exploratory trend analysis rather than confirmatory statistical inference. Significance testing (p‑values) was not performed due to insufficient degrees of freedom; these coefficients serve to visualize co‑variation patterns among instrumental and sensory variables across the storage timeline.

**Table S4.** Volatile compounds at different storage times of pork with salted vegetable.

| **No.** | **Compound** | **CAS#** | **Formula** | **MW** | **RI** | **Rt** | **Dt** | **Relative content** | | |
| --- | --- | --- | --- | --- | --- | --- | --- | --- | --- | --- |
|  |  |  |  |  |  |  |  | **G** | **O** | **T** |
| 1 | n-Nonanal | C124196 | C9H18O | 142.2 | 1103.8 | 783.65 | 1.4699 | 180.01±8.13^a^ | 162.97±5.07^a^ | 160.98±9.45^a^ |
| 2 | (E)-2-Octenal | C2548870 | C8H14O | 126.2 | 1065.9 | 703.427 | 1.32779 | 164.64±11.37^c^ | 236.19±12.11^b^ | 284.99±19.32^a^ |
| 3 | 1-Octanol | C111875 | C8H18O | 130.2 | 1065.9 | 703.427 | 1.45378 | 48.9±8.11^c^ | 65.17±1.43^b^ | 81.1±5.27^a^ |
| 4 | 2-Phenylacetaldehyde monomer | C122781 | C8H8O | 120.2 | 1052.9 | 677.714 | 1.25601 | 1166.47±64.36^a^ | 787.81±33.18^b^ | 573.63±54.77^c^ |
| 5 | 2-Phenylacetaldehyde dimer | C122781 | C8H8O | 120.2 | 1052.9 | 677.714 | 1.53729 | 221.1±19.96^a^ | 108.25±9.35^b^ | 62.56±11.28^c^ |
| 6 | 1,8 Cineole | C470826 | C10H18O | 154.3 | 1039.3 | 651.936 | 1.29334 | 40.38±1.52^b^ | 66.06±2.24^a^ | 49.54±6.11^b^ |
| 7 | (E,E)-2,4-Heptadienale monomer | C4313035 | C7H10O | 110.2 | 1022.1 | 620.858 | 1.19708 | 116.38±5.81^c^ | 256.24±4.66^b^ | 441.4±14.45^a^ |
| 8 | (E,E)-2,4-Heptadienal dimer | C4313035 | C7H10O | 110.2 | 1021.8 | 620.238 | 1.61953 | 23.7±1.22^c^ | 32.91±2.67^b^ | 46.42±4.84^a^ |
| 9 | n-Octanale monomer | C124130 | C8H16O | 128.2 | 1010 | 599.766 | 1.40926 | 541.08±21.28^b^ | 579.63±16.37^b^ | 669.81±16.06^a^ |
| 10 | n-Octanal dimer | C124130 | C8H16O | 128.2 | 1011.1 | 601.627 | 1.81833 | 31.52±3.06^b^ | 36.28±0.58^b^ | 46.01±3.05^a^ |
| 11 | Ethyl hexanoate | C123660 | C8H16O2 | 144.2 | 1007.8 | 596.044 | 1.33662 | 171.46±7.55^a^ | 104.2±4.4^b^ | 94.42±6.3^b^ |
| 12 | Benzaldehydee monomer | C100527 | C7H6O | 106.1 | 963.5 | 507.332 | 1.15311 | 1165.87±49.09^a^ | 1208.58±29.82^a^ | 938.35±10.07^b^ |
| 13 | Benzaldehyde dimer | C100527 | C7H6O | 106.1 | 963.2 | 506.711 | 1.47234 | 560.69±80.42^a^ | 667.11±54.65^a^ | 381.57±8.2^b^ |
| 14 | (E)-2-Heptenale monomer | C18829555 | C7H12O | 112.2 | 958.1 | 496.786 | 1.25443 | 906.74±102.18^c^ | 1287.6±43.56^b^ | 1729.87±69.76^a^ |
| 15 | (E)-2-Heptenal dimer | C18829555 | C7H12O | 112.2 | 958.1 | 496.786 | 1.67688 | 267.85±41.66^c^ | 548.52±45.72^b^ | 1068.09±125.53^a^ |
| 16 | 2-Pentyl furan | C3777693 | C9H14O | 138.2 | 995 | 573.711 | 1.25634 | 138.84±5.89^b^ | 162.73±6.24^a^ | 71.47±4.5^c^ |
| 17 | 1-Octen-3-onee monomer | C4312996 | C8H14O | 126.2 | 981.3 | 543.933 | 1.27737 | 119.42±1.04^b^ | 153.03±11.21^b^ | 336.94±22.45^a^ |
| 18 | 1-Octen-3-one dimer | C4312996 | C8H14O | 126.2 | 981.9 | 545.174 | 1.6807 | 29.13±2.95^b^ | 29.88±2.11^b^ | 43.29±4.23^a^ |
| 19 | Acetone | C67641 | C3H6O | 58.1 | 505.1 | 111.732 | 1.12937 | 4324.09±209.26^b^ | 4970.09±183.28^a^ | 3342.64±104.35^c^ |
| 20 | 2-Butanonee monomer | C78933 | C4H8O | 72.1 | 582.5 | 137.229 | 1.08703 | 1784.62±306.99^a^ | 1740.29±392.29^a^ | 2082.15±72.95^a^ |
| 21 | 2-Butanone dimer | C78933 | C4H8O | 72.1 | 585.8 | 138.454 | 1.24988 | 5243.45±324.83^a^ | 5467.07±1334.47^a^ | 3561.12±1375.5^a^ |
| 22 | Acetic acid ethyl estere monomer | C141786 | C4H8O2 | 88.1 | 612.8 | 148.751 | 1.10766 | 771.48±58.16^a^ | 485.5±46.05^b^ | 474.05±7.86^b^ |
| 23 | Acetic acid ethyl ester dimer | C141786 | C4H8O2 | 88.1 | 609.7 | 147.525 | 1.33782 | 1137.94±38.55^a^ | 335.8±36.17^b^ | 185.08±38.44^c^ |
| 24 | 2-Methylpropanal | C78842 | C4H8O | 72.1 | 597.6 | 142.867 | 1.29005 | 1897.98±35.26^a^ | 1031.42±94.77^b^ | 698.36±143.22^c^ |
| 25 | 2-Methyl Butanale monomer | C96173 | C5H10O | 86.1 | 667.6 | 172.041 | 1.17497 | 670.37±82.67^a^ | 595.59±55.31^a^ | 669.86±24.43^a^ |
| 26 | 2-Methyl Butanal dimer | C96173 | C5H10O | 86.1 | 664.3 | 170.57 | 1.40079 | 2233.68±114.54^a^ | 1060.66±235.92^b^ | 594.02±152.06^c^ |
| 27 | 3-Methyl butanale monomer | C590863 | C5H10O | 86.1 | 644.3 | 161.744 | 1.19994 | 1464.6±111.2^b^ | 1502.24±71.3^b^ | 1711.35±68.65^a^ |
| 28 | 3-Methyl butanal dimer | C590863 | C5H10O | 86.1 | 648.9 | 163.706 | 1.40839 | 2490.41±183.66^a^ | 1555.59±368.99^b^ | 762±216.23^c^ |
| 29 | Pentanale monomer | C110623 | C5H10O | 86.1 | 696.5 | 187.486 | 1.20211 | 714.98±80.59a^b^ | 437.92±100.99^a^ | 760.39±153.47^a^ |
| 30 | Pentanal dimer | C110623 | C5H10O | 86.1 | 697.5 | 188.221 | 1.42685 | 437.16±119.77^a^ | 413.57±138.02^a^ | 527.98±186.39^a^ |
| 31 | Acetoine monomer | C513860 | C4H8O2 | 88.1 | 711.5 | 198.518 | 1.06966 | 1708.65±117.32^a^ | 1935.34±135.2^a^ | 1696.19±182.83^a^ |
| 32 | Acetoin dimer | C513860 | C4H8O2 | 88.1 | 713.4 | 199.989 | 1.33131 | 594.25±36.67^b^ | 1144.73±89.24^c^ | 441.72±12.61^a^ |
| 33 | 1-Hydroxy-2-propanonee monomer | C116096 | C3H6O2 | 74.1 | 684.9 | 180.131 | 1.06857 | 1568.45±174.53^a^ | 1615.8±280.27^a^ | 1259.24±286.28^a^ |
| 34 | 1-Hydroxy-2-propanone dimer | C116096 | C3H6O2 | 74.1 | 683.8 | 179.641 | 1.23794 | 703.33±20.53^b^ | 1031.33±31.19^a^ | 570.11±27.03^c^ |
| 35 | (E)-2-Pentenale monomer | C1576870 | C5H8O | 84.1 | 748.3 | 228.427 | 1.10983 | 455.85±19.37^c^ | 638.32±5.12^b^ | 932.57±12.88^a^ |
| 36 | (E)-2-Pentenal dimer | C1576870 | C5H8O | 84.1 | 748.3 | 228.427 | 1.36388 | 78.14±17.61^c^ | 192.51±17.33^b^ | 568.03±21.15^a^ |
| 37 | 3-Penten-2-onee monomer | C625332 | C5H8O | 84.1 | 738.5 | 220.092 | 1.09897 | 330.84±3.9^a^ | 299.6±7.04^b^ | 274.03±3.33^c^ |
| 38 | 3-Penten-2-one dimer | C625332 | C5H8O | 84.1 | 736.7 | 218.621 | 1.34217 | 134.66±6.07^b^ | 237.5±7.58^a^ | 108.14±2.89^c^ |
| 39 | 1-Pentanole monomer | C71410 | C5H12O | 88.1 | 760.3 | 239.214 | 1.25857 | 757.22±12.94^b^ | 826.54±18.63^a^ | 819.92±11.52^a^ |
| 40 | 1-Pentanol dimer | C71410 | C5H12O | 88.1 | 760.6 | 239.459 | 1.51479 | 172.48±5.58^b^ | 247.55±17.19^a^ | 221.31±10.63^a^ |
| 41 | (Z)-2-Penten-1-ol | C1576950 | C5H10O | 86.1 | 766.2 | 244.607 | 0.94806 | 102.15±10.26^c^ | 201.93±6.81^b^ | 275.48±3.39^a^ |
| 42 | 1-Penten-3-ol | C616251 | C5H10O | 86.1 | 674.3 | 175.173 | 0.94242 | 124.76±2.53^b^ | 168.66±28.9^b^ | 347.96±49.25^a^ |
| 43 | 3-Methyl-2-butenale monomer | C107868 | C5H8O | 84.1 | 776.7 | 254.647 | 1.09512 | 617.68±5.63^a^ | 516.44±3.19^b^ | 453.46±27.65^c^ |
| 44 | 3-Methyl-2-butenal dimer | C107868 | C5H8O | 84.1 | 776.3 | 254.213 | 1.35936 | 171.92±4.84^a^ | 122.91±15.97^b^ | 79.3±1.96^c^ |
| 45 | 1-Hexanale monomer | C66251 | C6H12O | 100.2 | 792.1 | 269.413 | 1.26641 | 1655.66±39.47^a^ | 1496.35±26.93^b^ | 1651.59±31.74^a^ |
| 46 | 1-Hexanal dimer | C66251 | C6H12O | 100.2 | 791.1 | 268.544 | 1.56384 | 3222.43±24.44^b^ | 3583.89±109.54^a^ | 3603.35±150.94^a^ |
| 47 | Furfurale monomer | C98011 | C5H4O2 | 96.1 | 830.7 | 308.933 | 1.08583 | 1763.11±45.28^a^ | 1258.94±17.08^b^ | 1289.28±10.33^b^ |
| 48 | Furfural dimer | C98011 | C5H4O2 | 96.1 | 829.5 | 307.63 | 1.33944 | 1780.1±50.05^a^ | 885.19±9.39^b^ | 784.22±17.63^c^ |
| 49 | 3-Methylbutanol | C123513 | C5H12O | 88.1 | 728.8 | 212.087 | 1.2518 | 320.06±5.34^a^ | 313.91±14.57^a^ | 213.75±19^b^ |
| 50 | 2-Hexenale monomer | C505577 | C6H10O | 98.1 | 849.1 | 329.779 | 1.18541 | 359.36±44.92^c^ | 514.95±12.9^b^ | 737.7±6.07^a^ |
| 51 | 2-Hexenal dimer | C505577 | C6H10O | 98.1 | 849.5 | 330.213 | 1.51737 | 84.55±11.59^c^ | 184.07±1.24^b^ | 341.01±20.06^a^ |
| 52 | 1 |  |  |  | 858.6 | 341.07 | 0.95835 | 1506.91±52.78^a^ | 1422.99±41.63^a^ | 793.83±80.15^b^ |
| 53 | 2 |  |  |  | 857.5 | 339.768 | 1.31023 | 330.18±13.09^a^ | 270.6±6.56^b^ | 79.88±11.72^c^ |
| 54 | 3 |  |  |  | 841.6 | 321.093 | 1.21861 | 271.53±37.49^a^ | 302.44±29.99^a^ | 173.22±32.31^b^ |
| 55 | Heptanale monomer | C111717 | C7H14O | 114.2 | 900.4 | 396.684 | 1.34253 | 616.96±10.37^b^ | 716.37±19.69^a^ | 742±17.93^a^ |
| 56 | Heptanal dimer | C111717 | C7H14O | 114.2 | 901 | 397.575 | 1.69847 | 118.88±6.65^b^ | 194.67±10.7^a^ | 178.07±3.33^a^ |
| 57 | 2-Heptanonee monomer | C110430 | C7H14O | 114.2 | 890.5 | 381.971 | 1.26636 | 341.92±9.24^b^ | 376.59±12.17^a^ | 317.32±5.13^c^ |
| 58 | 2-Heptanone dimer | C110430 | C7H14O | 114.2 | 889.5 | 380.634 | 1.63629 | 45.76±3.43^b^ | 59.4±1.81^a^ | 35.59±2.69^c^ |
| 59 | Cyclohexanone | C108941 | C6H10O | 98.1 | 898.1 | 393.117 | 1.1529 | 82.95±4.97^c^ | 126.1±2.81^b^ | 177.52±6.76^a^ |
| 60 | Isoamyl acetatee monomer | C123922 | C7H14O2 | 130.2 | 875.7 | 362.355 | 1.30522 | 369.9±6.86^a^ | 163.2±4.25^b^ | 129.4±3.01^c^ |
| 61 | Isoamyl acetate dimer | C123922 | C7H14O2 | 130.2 | 876.4 | 363.246 | 1.74821 | 46.7±6.19^a^ | 16.58±0.73^b^ | 13.37±3^b^ |
| 62 | 3-Methylthiopropanale monomer | C3268493 | C4H8OS | 104.2 | 907.5 | 407.829 | 1.09228 | 542.32±7.36^a^ | 350.46±22.19^b^ | 225.12±7.58^c^ |
| 63 | 3-Methylthiopropanal dimer | C3268493 | C4H8OS | 104.2 | 907.2 | 407.384 | 1.40004 | 96.8±3.71^a^ | 65.13±8.49^b^ | 30.81±3.07^c^ |
| 64 | 4 |  |  |  | 869 | 353.884 | 1.1016 | 330.41±5.15^b^ | 376.7±14.91^a^ | 296.74±17.55^b^ |
| 65 | 1-Hexanol | C111273 | C6H14O | 102.2 | 870.1 | 355.221 | 1.32854 | 80.51±5.1^b^ | 95.66±1.56^a^ | 63.44±2.85^c^ |
| 66 | 4-Heptanone | C123193 | C7H14O | 114.2 | 874.3 | 360.571 | 1.22906 | 151.32±3.72^a^ | 92.82±4.54^b^ | 60.34±3.6^c^ |
| 67 | Ethyl 2-methylbutanoatee monomer | C7452791 | C7H14O2 | 130.2 | 845.9 | 326.112 | 1.24698 | 263.58±26.77^a^ | 57.23±7.61^b^ | 57.04±4.3^b^ |
| 68 | Ethyl 2-methylbutanoate dimer | C7452791 | C7H14O2 | 130.2 | 845.8 | 325.905 | 1.64667 | 54.95±7.69^a^ | 15.69±0.52^b^ | 12.5±4.38^b^ |
| 69 | 2,5-Dimethylpyrazine | C123320 | C6H8N2 | 108.1 | 914 | 418.402 | 1.11834 | 180.87±4.56^a^ | 159.61±1.53^b^ | 156.48±12.58^b^ |
| 70 | Gamma-Butyrolactone | C96480 | C4H6O2 | 86.1 | 921.9 | 431.363 | 1.08642 | 345.77±10.5^a^ | 389.74±23.92^a^ | 354.95±36.61^a^ |
| 71 | 5 |  |  |  | 948.1 | 477.833 | 1.2571 | 39.93±3.16^c^ | 63.2±4.54^b^ | 99.57±6.83^a^ |
| 72 | 6 |  |  |  | 961.3 | 503.123 | 1.56932 | 209.54±34.64^b^ | 352.85±21.37^a^ | 394.91±12.12^a^ |
| 73 | Methyl-5-hepten-2-one | C110930 | C8H14O | 126.2 | 990.1 | 562.887 | 1.18047 | 203.65±11.26^c^ | 323.88±6.88^a^ | 279±5.36^b^ |
| 74 | 1-Octen-3-ol | C3391864 | C8H16O | 128.2 | 985.1 | 551.915 | 1.16096 | 323.64±13.03^b^ | 447.97±14.46^a^ | 469.21±19.09^a^ |
| 75 | 7 |  |  |  | 988 | 558.185 | 1.43224 | 212.75±21.51^b^ | 222.85±6.83^b^ | 359.88±36.18^a^ |
| 76 | Alpha-Phellandrene | C99832 | C10H16 | 136.2 | 1002.6 | 587.181 | 1.20884 | 39.72±0.84^b^ | 55.65±3.87^b^ | 120.09±19.28^a^ |
| 77 | Isopentyl propanoate | C105680 | C8H16O2 | 144.2 | 970.5 | 521.351 | 1.33472 | 86.3±5.35^a^ | 46.97±2.08^b^ | 24.06±2.61^c^ |
| 78 | Beta-Ocimene | C13877913 | C10H16 | 136.2 | 1041.5 | 656.145 | 1.26557 | 72.89±3.76^b^ | 100.54±3.03^a^ | 103.51±3.94^a^ |
| 79 | Isoamyl butyrate | C106274 | C9H18O2 | 158.2 | 1044.9 | 662.415 | 1.42337 | 56.47±2.58^c^ | 71.37±4.17^b^ | 100.39±6.46^a^ |

Note: Data are presented as mean ± standard deviation(n=3). Mean values with different letters indicate significant differences(p<0.05) in the volatile compound amounts between storage periods, as determined by ANOVA followed by Duncan’s multiple comparison test. G represents the fresh sample, O represents the sample stored for one month, and T represents the sample stored for two months.

**Table S5.** ROAV of pork with salted vegetable.

| **Compound** | **Flavor description** | Odour  Threshold Value | ROAV(%) | | |
| --- | --- | --- | --- | --- | --- |
|  |  |  | G | O | T |
| 1-Octen-3-one | strong earthy, mushroom, vegetable, fishy, chicken | 0.005 | 100.0000 | 100.0000 | 100.0000 |
| 1-Hexanal | fresh, green, fat, fruity | 1.4 | 14.5890 | 11.8562 | 5.5700 |
| 2-Methyl Butanal | almond, cocoa, malt | 1 | 12.1593 | 5.4115 | 1.8755 |
| 3-Methyl butanal | chocolate, fat | 1.6 | 10.3498 | 6.2443 | 2.2939 |
| 2-Phenylacetaldehyde | hyacinth, sweet fruity, almond, cherry, clover honey, cocoa | 0.6 | 9.6830 | 4.8795 | 1.5734 |
| Heptanal | fresh, aldehyde, fatty, green herbs, wine, fruity | 0.85 | 3.6247 | 3.5019 | 1.6063 |
| Pentanal | green grassy, faint banana, pungent | 1.4 | 3.4457 | 1.9872 | 1.3656 |
| n-Octanal | aldehyde, waxy, citrus, orange, fruity, fatty | 0.88 | 2.7244 | 2.2868 | 1.2071 |
| n-Nonanal | rose, citrus, strong oily | 0.3 | 2.5123 | 1.7749 | 0.7963 |
| 3-Penten-2-one | Fruity, turns into spicy during storage | 1.5 | 1.2994 | 1.1699 | 0.3781 |
| 1-Octen-3-ol | mushroom, lavender, rose, hay | 1.5 | 0.9034 | 0.9758 | 0.4642 |
| 2-Methylpropanal | banana, melon , slightly nutty | 10 | 0.7947 | 0.3370 | 0.1036 |
| Acetoin | butter, cream | 14 | 0.6887 | 0.7188 | 0.2266 |
| 2-Hexenal | sweet almonds, fruity, green leaves, apples, plums, vegetables | 3.1 | 0.5996 | 0.7367 | 0.5164 |
| 1-Penten-3-ol | ethereal, green, tropical fruity | 1 | 0.5224 | 0.5511 | 0.5163 |
| 2-Heptanone | pear, banana, fruity, slight medicinal fragrance | 3.5 | 0.4638 | 0.4070 | 0.1496 |
| (E)-2-Heptenal | spicy, green vegetables, fresh, fatty | 13 | 0.3783 | 0.4615 | 0.3194 |
| (E)-2-Octenal | fresh cucumber, fatty, green herbal, banana, green leaf | 3 | 0.2298 | 0.2572 | 0.1410 |
| Isoamyl acetate | sweet, banana, fruity | 7 | 0.2213 | 0.0762 | 0.0274 |
| 4-Heptanone | Fruity | 8.2 | 0.0773 | 0.0370 | 0.0109 |
| 1,8 Cineole | camphor, refreshing herbal | 2.67 | 0.0633 | 0.0808 | 0.0275 |
| Benzaldehyde | bitter almond, cherry, nutty | 85 | 0.0503 | 0.0460 | 0.0196 |
| Methyl-5-hepten-2-one | citrus, fruity, mouldy, ketone | 17 | 0.0502 | 0.0622 | 0.0244 |
| γ-Butyrolactone | cream, fat, caramel | 35 | 0.0414 | 0.0364 | 0.0150 |
| (E,E)-2,4-Heptadienal | fatty, oily, aldehyde, vegetable, cinnamon | 15.4 | 0.0381 | 0.0613 | 0.0470 |
| Isoamyl butyrate | (bananas, pears | 7 | 0.0338 | 0.0333 | 0.0213 |
| 2-Pentyl furan | bean, fruity, earthy, green, vegetable | 19 | 0.0306 | 0.0280 | 0.0056 |
| 1-Pentanol | balsamic | 153 | 0.0254 | 0.0229 | 0.0101 |
| Ethyl 2-methylbutanoate | apple | 58 | 0.0230 | 0.0041 | 0.0018 |
| Isopentyl propanoate | fruity | 17 | 0.0213 | 0.0090 | 0.0021 |
| 1-Hexanol | fresh, fruity, wine, sweet, green | 34 | 0.0099 | 0.0092 | 0.0028 |
| Acetone | fresh, apple, pear | 2000 | 0.0091 | 0.0081 | 0.0025 |
| Beta-Ocimene | floral, woody, fruity | 34 | 0.0090 | 0.0097 | 0.0045 |
| 1-Octanol | citrus, sweet, herbs, waxy, rose, mushroom | 23 | 0.0089 | 0.0093 | 0.0052 |
| Furfural | sweet, woody, almond, bready | 2800 | 0.0053 | 0.0025 | 0.0011 |
| 2,5-Dimethylpyrazine | nutty, peanut, mouldy, earthy, potato, fatty, cocoa powder | 170 | 0.0045 | 0.0031 | 0.0014 |
| (E)-2-Pentenal | potato, peas | 1400 | 0.0016 | 0.0019 | 0.0016 |
| Cyclohexanone | strong pungent, earthy | 1100 | 0.0003 | 0.0004 | 0.0002 |
| Alpha-Phellandrene | dill | 2900 | 0.0001 | 0.0001 | 0.0001 |
| Ethyl hexanoate | pineapple, fruity, wine | 13200 | 0.0001 | 0.0000 | 0.0000 |

**Table S6.** VIP scores of pork with salted vegetable.

| **Var ID (Primary)** | **M2.VIP[2]** |
| --- | --- |
| **3-Penten-2-one dimer** | 1.46895 |
| **Acetoin dimer** | 1.44969 |
| **1-Hydroxy-2-propanone dimer** | 1.44297 |
| **1-Hexanal monomer** | 1.36574 |
| **2-Heptanone dimer** | 1.35257 |
| **1,8 Cineole** | 1.34991 |
| **2-Heptanone monomer** | 1.31668 |
| **1-Hexanol** | 1.30688 |
| **4** | 1.2994 |
| **Methyl-5-hepten-2-one** | 1.28155 |
| **Acetone** | 1.26866 |
| **2-Pentyl furan** | 1.21269 |
| **1-Pentanol dimer** | 1.19699 |
| **Heptanal dimer** | 1.17168 |
| **Benzaldehyde dimer** | 1.16826 |
| **Pentanal monomer** | 1.1543 |
| **Benzaldehyde monomer** | 1.11674 |
| **Furfural monomer** | 1.1001 |
| **Ethyl 2-methylbutanoate monomer** | 1.0616 |
| **3** | 1.03551 |
| **Furfural dimer** | 1.02243 |
| **1-Pentanol monomer** | 1.02104 |
| **Acetic acid ethyl ester monomer** | 1.00891 |
| **Isoamyl acetate monomer** | 1.00386 |
| **Ethyl 2-methylbutanoate dimer** | 1.00376 |
| **3-Methylbutanol** | 1.00194 |
| **Ethyl hexanoate** | 0.995526 |
| **1** | 0.995146 |
| **Beta-Ocimene** | 0.993756 |
| **Isoamyl acetate dimer** | 0.99306 |
| **Acetic acid ethyl ester dimer** | 0.992089 |
| **1-Octen-3-one monomer** | 0.983227 |
| **7** | 0.974396 |
| **1-Octen-3-ol** | 0.973381 |
| **(E)-2-Pentenal dimer** | 0.953411 |
| **2** | 0.950357 |
| **1-Octen-3-one dimer** | 0.937412 |
| **Heptanal monomer** | 0.930739 |
| **Alpha-Phellandrene** | 0.92506 |
| **2-Methylpropanal** | 0.922946 |
| **1-Penten-3-ol** | 0.921701 |
| **6** | 0.920218 |
| **2-Phenylacetaldehyde dimer** | 0.91619 |
| **2-Methyl Butanal dimer** | 0.909639 |
| **4-Heptanone** | 0.908258 |
| **(E)-2-Pentenal monomer** | 0.902703 |
| **Isopentyl propanoate** | 0.90122 |
| **3-Methylthiopropanal monomer** | 0.898191 |
| **2-Hexenal dimer** | 0.896508 |
| **(E,E)-2,4-Heptadienal monomer** | 0.893724 |
| **1-Hexanal dimer** | 0.893006 |
| **(Z)-2-Penten-1-ol** | 0.892999 |
| **Acetoin monomer** | 0.892379 |
| **2-Phenylacetaldehyde monomer** | 0.891928 |
| **Isoamyl butyrate** | 0.887953 |
| **(E)-2-Heptenal dimer** | 0.88738 |
| **2-Hexenal monomer** | 0.885515 |
| **Cyclohexanone** | 0.885262 |
| **5** | 0.884547 |
| **n-Octanal monomer** | 0.882187 |
| **3-Methyl-2-butenal monomer** | 0.880705 |
| **3-Penten-2-one monomer** | 0.873961 |
| **3-Methylthiopropanal dimer** | 0.872635 |
| **Gamma-Butyrolactone** | 0.870851 |
| **(E)-2-Heptenal monomer** | 0.870661 |
| **3-Methyl-2-butenal dimer** | 0.864358 |
| **(E)-2-Octenal** | 0.863823 |
| **(E,E)-2,4-Heptadienal dimer** | 0.849531 |
| **n-Octanal dimer** | 0.849244 |
| **3-Methyl butanal dimer** | 0.834892 |
| **2,5-Dimethylpyrazine** | 0.821518 |
| **1-Octanol** | 0.818945 |
| **3-Methyl butanal monomer** | 0.780404 |
| **2-Methyl Butanal monomer** | 0.764085 |
| **n-Nonanal** | 0.756915 |
| **2-Butanone dimer** | 0.686562 |
| **1-Hydroxy-2-propanone monomer** | 0.60849 |
| **2-Butanone monomer** | 0.529517 |
| **Pentanal dimer** | 0.370392 |
